# An evaluation of the Mitsubishi GL-101 glucose analyser

**DOI:** 10.1155/S1463924684000055

**Published:** 1984

**Authors:** Susan Oldfield, A. E. Hurrell, J. D. McVittie, D. Quantrill

**Affiliations:** 1 Department of Biochemistry Good Hope General Hospital West Midlands Sutton Coldfield B75 7RR UK; 2 Department of Clinical Biochemistry John Radcliffe Hospital Headington Oxford OX3 9DU UK


Journal of Automatic Chemistry, Volume 6 Number 1 (January–March 1984), pages 21–26

### An evaluation of the Mitsubishi GL-101 glucose analyser

**Susan Oldfield, A. E. Hurrell**

*Department of Biochemistry, Good Hope General Hospital, Sutton Coldfield, West Midlands B75 7RR, UK*

**J. D. McVittie and D. Quantrill**

*Department of Clinical Biochemistry, John Radcliffe Hospital, Headington, Oxford OX3 9DU, UK*

### Introduction

The GL-101 Glucose Analyser is manufactured in Japan by Mitsubishi Chemical Industries Ltd, and marketed in the UK by Anachem Ltd. A prototype machine was lent to the John Radcliffe Hospital, Oxford, during late 1982 and a modified model was installed in Good Hope Hospital, Sutton Coldfield, in early January 1983.

The analyser is designed solely for the estimation of glucose with sampling, calibration and production of result being completely automatic. It has the ability to analyse both plasma and urine samples and the sampling cycle can be interrupted at any time to accommodate emergency samples. The instrument is approximately 50 cm high, 50 cm wide and 50 cm long and the only service required is a single electric output socket, although it is more convenient to site the analyser near a drain to take the waste solutions from the instrument. The sampler has a 40-position tray which accepts standard analyser cups and has an optional sample-identification system utilizing coded cards which fit over the cups. Sampling, dilution with water and the addition of phosphate buffer is achieved by means of automatic syringes. The diluted sample is pumped through a pretreatment column of basic anion-exchange resin, to remove possible interfering substances, before the addition of buffer and passage through a column of glucose oxidase (GOD) immobilized on glass beads. The manufacturer claims that 2000 samples can be passed through the pretreatment column and 500 samples through the GOD column before replacement is necessary. The amount of hydrogen peroxide formed is proportional to the glucose present and is measured by an electrode system positioned immediately above the enzyme column.

By identification of the cup containing the standard and the value of the standard, the analyser will calibrate itself and calculate and print-out the values of test samples. However, it is possible to override the manufacturer's standard value and enter any other positive integer.

The instrument samples at up to 120 samples/h utilizing 6 $\mu$l of plasma (although requiring 20 $\mu$l in the sample cup) or 2 $\mu$l of urine. Results are printed on paper on a 24-column printer, in addition to being displayed by the LED screen; an RS 232C unidirectional interface card is available as an optional extra to allow direct transmission of results to a main laboratory computer.

### Safety

The only moving parts accessible to the operator are the sampler probe and turntable. The small distance of travel of the sampler probe, facilitated by the compact design of the machine, is unlikely to present a hazard to the operator. The only reagents required are phosphate buffer and water and spillage of these onto the electronic components is unlikely.

### *Microbiological safety*

The sampler area is easily cleaned but it is difficult to disinfect the tubing. Dissemination of infective material was assessed as recommended by Broughton *et al.* [1]. Plates containing culture medium were placed on and around the machine, a tray of 40 aliquots of a concentrated suspension of *Bacillus globigii* was sampled and analysed as serum and the plates then incubated for three days. No growth of the *Bacillus* was found on any plate and air sampled from the vicinity of the sample probe showed no contamination. At the end of the run a sample of water was taken for culture from the rinse cuvette and the columns were removed, perfused with broth and incubated. No *Bacillus globigii* colonies were grown from the rinse water but, not surprisingly, the organism was cultured from both columns.

It is concluded that no significant aerosol formation or splashing occurs. The conditioning cycle at the end of each run does not remove trapped organisms from the columns. However, as the columns have a limited life, this should not be a problem; discarded columns should be treated as contaminated material.

### Analytical evaluation

### *Precision*

Serum pools containing low, medium and high concentrations of glucose were prepared from Gibco mycoplasma-screened horse serum. Glucose was added to give a final concentration of approximately 19 mmol/l for the high pool and approximately 10 mmol/l for the medium pool. No glucose addition was necessary for the low control as the horse serum contained approximately 3 mmol/l of endogenous glucose. The serum pools were divided into 8 ml aliquots and stored at −40°C.

To assess precision of the Mitsubishi GL-101 the following tray layout was used to include the effects of carry-over on precision [2]:

Low pool: Cup Nos. 9, 11, 14, 15, 20, 21, 24, 27, 28, 34 37, 39.
Medium pool: Cup Nos. 6, 7, 12, 16, 18, 19, 22, 23, 26, 30, 31, 33.
High pool: Cup Nos. 5, 8, 10, 13, 17, 25, 29, 32, 35, 36, 38, 40.

The instrument was calibrated at the start of each tray and control materials placed in cups 1 to 4. Identical trays were run on 40 occasions; once in the morning and once in the afternoon of 20 working days. Fresh aliquots of serum were thawed each day. For comparative purposes the tray was also analysed daily on the Beckman glucose analyser, this machine being re-calibrated every 10 samples to simulate normal operation.

Histograms of all results obtained in the precision study on the Mitsubishi GL-101 and Beckman glucose analyser are shown in figures 1 and 2 respectively. It is interesting that although the GL-101 purports to measure glucose concentration to two places of decimals it will only print certain numbers, for example a result for the medium pool may be 9·79, 9·83 or 9·89 but none of the intermediate values. The intervals correspond to approximately 1 mg/dl and the effect may be due to the electronic arithmetic package which was originally designed to produce results in mg/dl and subsequently converted to mmol/l. Figures 3 and 4 show the variation of the within-batch mean and within-batch standard deviation respectively, for each serum pool, obtained on both instruments throughout the period of study. The overall precision data is shown in table 1.

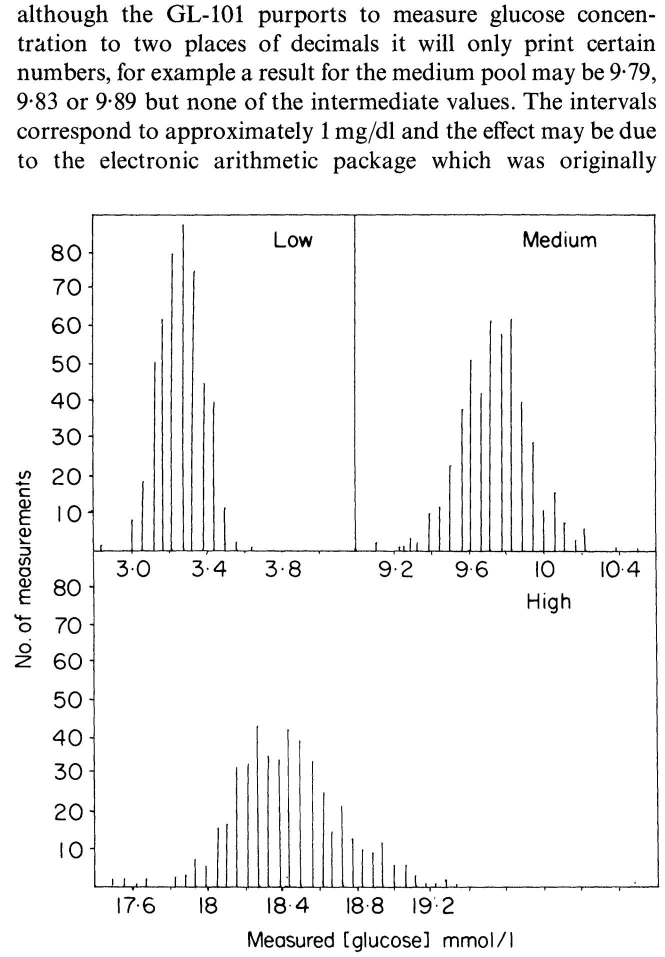

*Figure 1. Histograms of results obtained during the precision study on the Mitsubishi GL-101.*

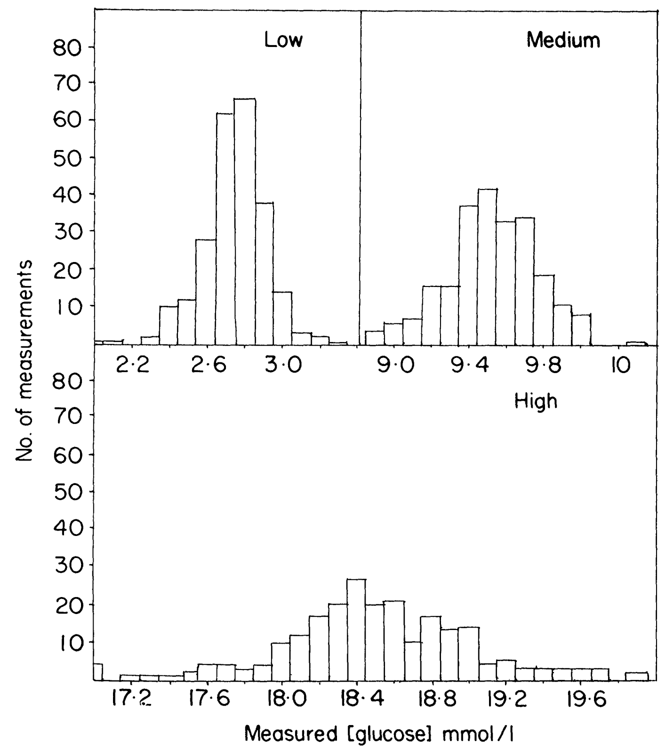

*Figure 2. Histograms of results obtained during the precision study on the Beckman glucose analyser.*

The precision of the GL-101 is adequate for normal, routine use and is superior to that of the Beckman glucose analyser which was formerly in routine operation at Good Hope Hospital. Mitsubishi quote a CV of better than 1% at a glucose concentration of 10 mmol/l; approximately half the authors' value. It appears that Mitsubishi's quoted CV is based on one run of identical specimens; therefore the authors' figure, which includes the effect of carry-over and day-to-day variations, is a better indication of the precision one would expect to obtain in routine operation. Analysis of 40 consecutive aliquots of a 10 mmol/l standard gave a mean value of 10·02 mmol/l, SD of 0·09 mmol/l and CV of 0·9, which meets the manufacturer's specification.

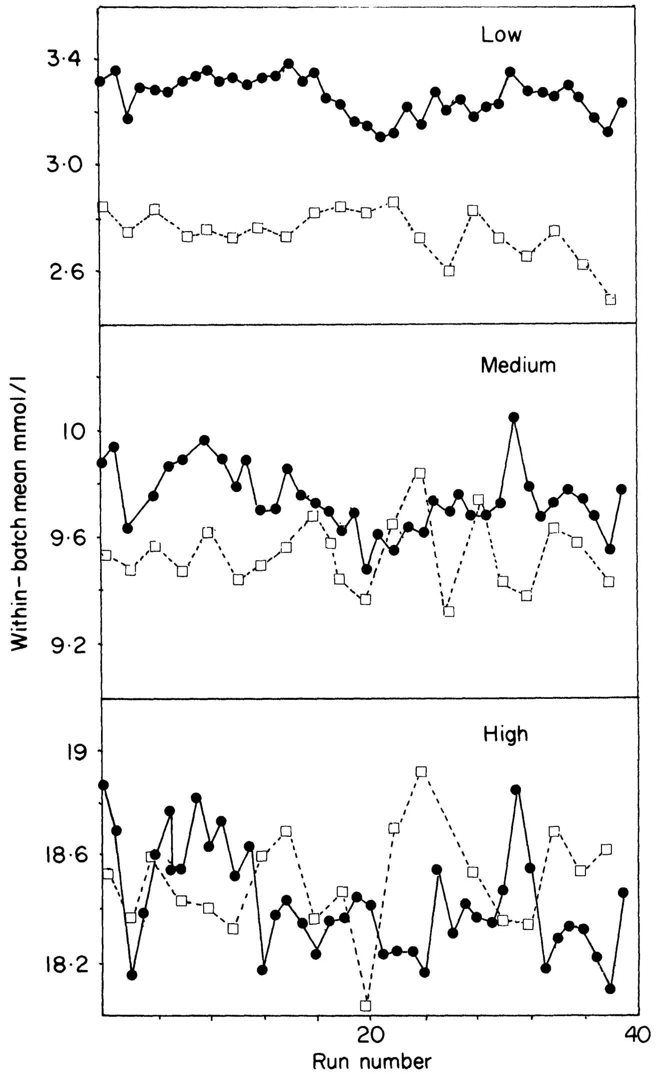

*Figure 3. Variation of within-batch mean for each serum pool during the precision study.* ●——● = *Mitsubishi GL-101,* □- - -□ = *Beckman glucose analyser.*

### *Comparison with other methods*

To assess the accuracy of the Mitsubishi GL-101 a selection of patients' samples obtained over a period of two weeks were analysed on both the Mitsubishi GL-101 and the Beckman glucose analyser. Results are shown in figure 5. The regression line was calculated for pairs of results less than 20 mmol/l and

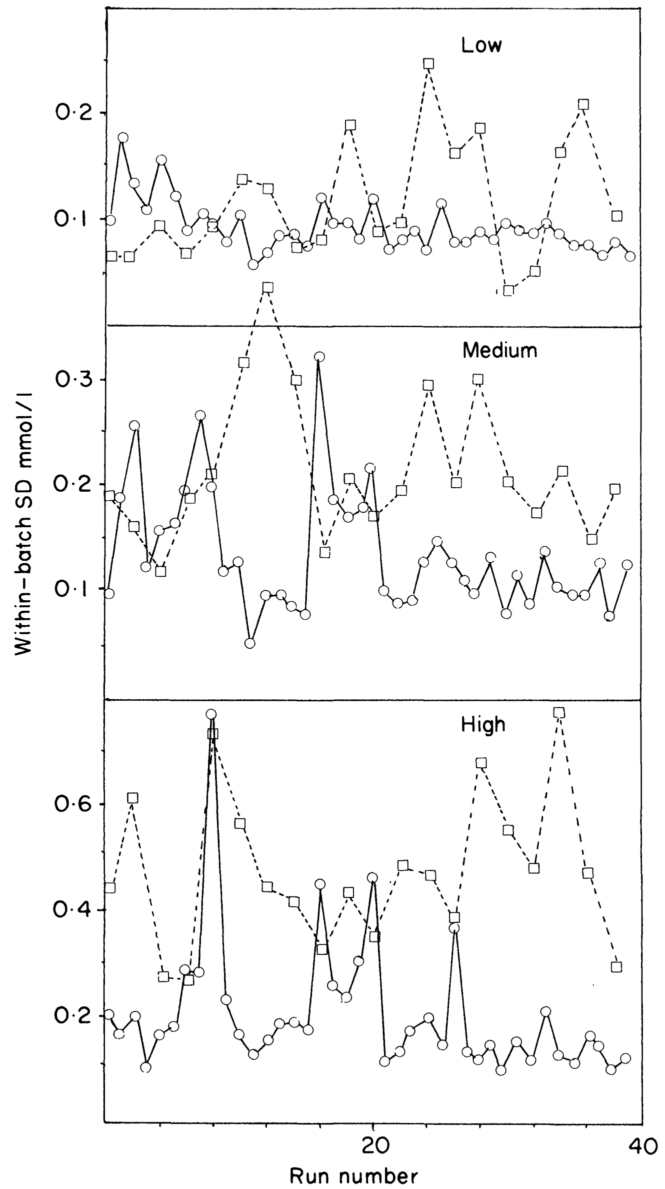

*Figure 4. Variation of within-batch standard deviation for each serum pool during the precision study.* ○——○ *= Mitsubishi GL-101,* □- - - -□ *= Beckman glucose analyser.*

found to have an intercept of 0·6 mmol/l and a gradient of 0·98. The higher results obtained using the Beckman machine for samples with a glucose concentration in excess of 23 mmol/l were subsequently shown to be a dilution error of double-filling the Beckman analytical cell with glucose oxidase reagent. A similar correlation study compared results of glucose analyses using the Mitsubishi GL-101 and the glucose oxidase (GOD PAP) method of BCL Ltd using a Gilford 3500 spectrophotometer; the latter method being in current use in the John Radcliffe Hospital.

Results are shown in figure 6 and the regression line had a gradient of 0·97 and an intercept of 0·18. The large positive bias of the Mitsubishi instrument at low glucose concentrations when compared to the Beckman machine is worrying. The effect may be partly explained by the sensitivity of the electrode to solutions containing protein (see below). The smaller intercept obtained in the second correlation may well be due to the different response of a different electrode to protein or other potentially interfering substances. Unfortunately it was impossible to obtain a new electrode system to permit further tests to be made before this publication.

| | | Mitsubishi GL-101 | Beckman |
|---|---|---|---|
| | Mean | 3·26 | 2·74 |
| Low pool | SD | 0·12 | 0·17 |
| | CV | 3·6 | 6·1 |
| | Mean | 9·74 | 9·53 |
| Medium pool | SD | 0·19 | 0·25 |
| | CV | 1·9 | 2·6 |
| | Mean | 18·43 | 18·50 |
| High pool | SD | 0·30 | 0·53 |
| | CV | 1·7 | 2·8 |

*Table 1. Precision data obtained on the Mitsubishi GL-101 and Beckman glucose analyser. See text for details.*

### *Linearity*

To investigate linearity of response of the GL-101 when set up to measure plasma glucose, a 50 mmol/l aqueous glucose standard was diluted with water to give a series of solutions with a range of glucose concentrations from 0 to 50 mmol/l. The instrument was calibrated with a 10 mmol/l standard and an identity flag to indicate the standard's concentration and each dilution was measured in duplicate, the series being run in both ascending and descending order. The GL-101 produced a linear response over the entire range tested (figure 7). Mitsubishi claim only that a linear response is obtained for samples with a glucose concentration of 40 mmol/l or less and an asterisk is printed alongside higher results to indicate their unreliability. It was subsequently found that the GL-101 would not print a result if the glucose concentration was greater than 60 mmol/l.

A second linearity experiment was performed with the instrument calibrated with a 30 mmol/l standard and the urine standard flag. An aqueous solution of approximately 500 mmol/l glucose was prepared and a series of dilutions made. The series was again run in ascending and descending order. Figure 8 shows that the response of the GL-101 under these conditions (sampling 2 $\mu$l) is linear up to a concentration of 250 mmol/l.

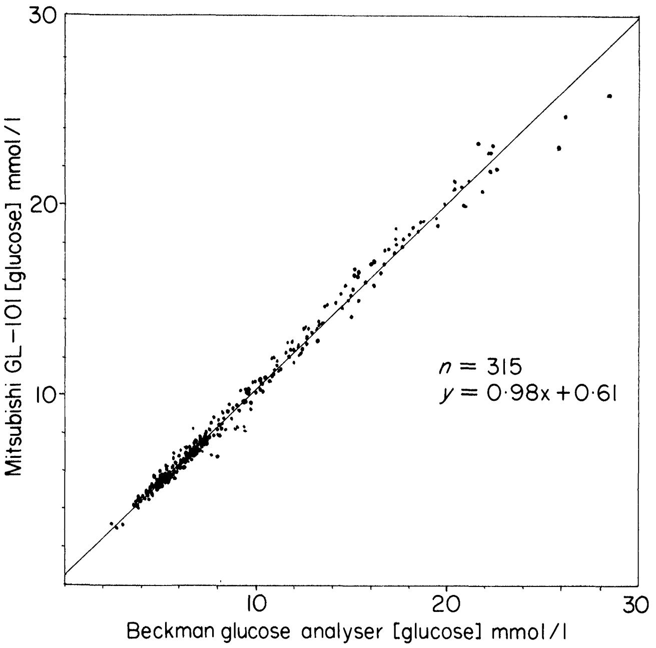

*Figure 5. Comparison of patients' results obtained on the Mitsubishi GL-101 with those obtained on the Beckman glucose analyser.*

### *Carry-over*

Carry-over was measured by the procedure of Broughton *et al.* [3]. Three successive aliquots of the high serum pool ($a_1, a_2, a_3$) were followed by three successive aliquots of the low serum pool ($b_1, b_2, b_3$).

Carry-over was calculated from the following formula:

$$\text{Carry-over} = \frac{b_1 - b_3}{a_3 - b_3} \times 100\%.$$

This procedure was carried out on five occasions on each of five days.

The mean carry-over was 1·21% and varied between 0·72% and 1·47%.

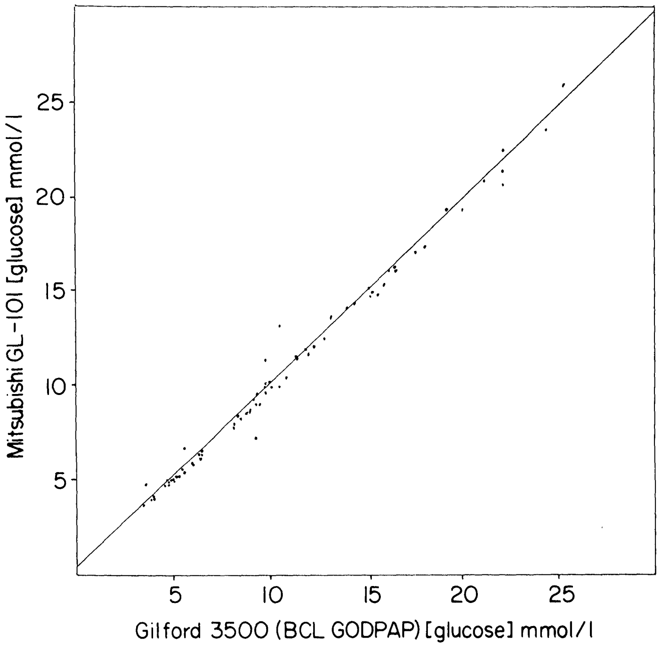

*Figure 6. Comparison of patients' results obtained on the Mitsubishi GL-101 with those obtained on the Gilford 3500 using the BCL GODPAP method.*

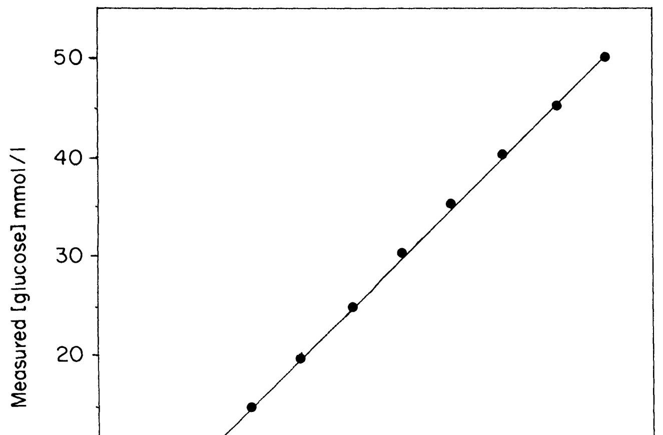

*Figure 7. Linearity of the Mitsubishi GL-101 standardized to measure plasma glucose.*

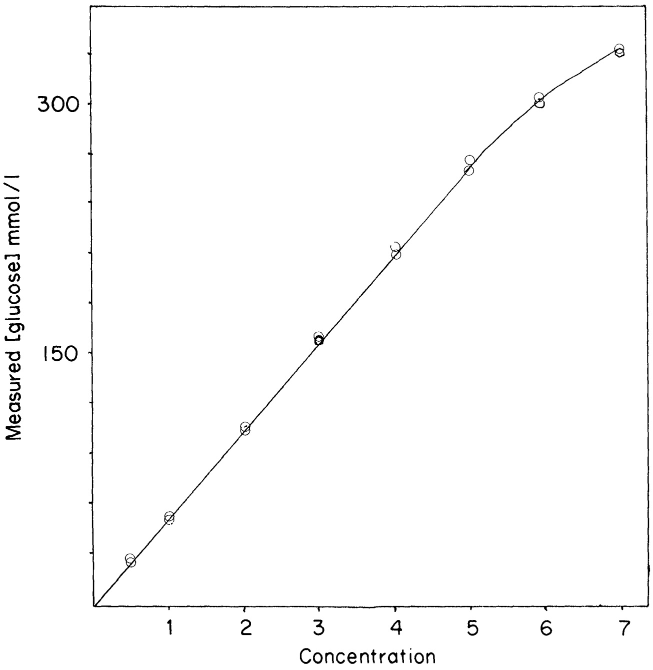

*Figure 8. Linearity of the Mitsubishi GL-101 standardized to measure urine glucose.*

### *Interfering substances*

Oxalate and fluoride, as anticoagulant and preservative respectively, are usually added to blood samples for glucose estimation. Sodium oxalate was added to aliquots of horse serum to give a range of concentrations up to 100 mmol/l, approximately eight times the normal concentration used. Similarly, potassium fluoride was added to horse serum to provide concentrations up to 330 mmol/l, about 15 times the concentration used to inhibit glycolysis. These and untreated samples were analysed on the GL-101. No interference was present at any concentration tested. However, a combination of 250 mmol/l fluoride and 250 mmol/l oxalate produced a slight positive interference (mean increase of 4·4% in apparent glucose concentration) on one Mitsubishi analyser, but had no effect on the other. Again, this may be due to a different response of the electrodes in the two machines but no replacement electrodes could be obtained to investigate this further. At the time of the study Anachem had few spare parts in the UK and, unfortunately, have subsequently decided to withdraw the instrument from the British market.

Ascorbic acid interference has been described in certain glucose methods [4], however, Mitsubishi claim that the pretreatment column removes ascorbate and other interfering substances from the assayed sample. Horse serum was supplemented with ascorbic acid to give a concentration of 200 mg/l, approximately 10 times the upper limit of normal plasma concentration in man, and glucose measurements were compared to those on untreated serum. No interference was observed.

It was postulated that haemolysis might interfere with glucose measurements by the GL-101 as this instrument measures hydrogen peroxide and haemoglobin has peroxidase activity. Haemolysis was simulated by the addition of erythrocyte lysate to horse serum to give a final haemoglobin concentration of approximately 20 g/l. Plasma (diluted to contain the same concentration of glucose as the lysate) was added to a second serum aliquot as a control, and water was added to a third. Each preparation was analysed five times: the mean measured glucose was 7·69 mmol/l in the haemolysed preparation, compared to 8·38 mmol/l in the control and

8·19 mmol/l in the sample to which water had been added. Therefore haemolysis causes a decrease in the apparent glucose concentration.

Interference by the analgesics salicylate and paracetamol was assessed by adding these drugs to horse serum. Paracetamol, in particular, has been found to interfere with some glucose methods [5]. Sodium salicylate was found to have no effect, but 500 mg/l of paracetamol caused an increase in apparent serum glucose of approximately 0·8 mmol/l. A dose-response curve using aqueous solutions of paracetamol showed a linear relationship up to at least 1 g/l (figure 9), however, the interference is not significant until high concentrations, only observed in very severe overdoses, are achieved.

To test the effect of urine preservatives, boric acid (2 g/l), thiomersal (0·2 g/l), 6M hydrochloric acid (10 ml/l) and benzoic acid (0·2 g/l) were added to separate aliquots of a urine known to contain glucose. The measured glucose concentration in each was compared to that of the untreated specimen; the effect in each case was not significant.

To test the effect of carbohydrates other than glucose, aliquots of a normal urine were supplemented with sucrose, lactose, galactose or xylose to a concentration of 2 g/l. These sugars did not interfere with glucose measurements.

A possible explanation for the consistently higher results obtained on the Mitsubishi instrument compared to the Beckman glucose analyser (see before) could be interference from protein. To test this hypothesis, 10 mmol/l and 50 mmol/l glucose standards were diluted 10 times into saline or solutions of human serum albumin or Sigma standard protein solution (50 g/l human albumin, 30 g/l human globulin) in saline and measured glucose concentrations were compared. Protein appears to interfere (results shown in table 2) with the GL-101 giving a result of almost 0·4 mmol/l for a solution 80 g/l human serum proteins. This is rather worrying, especially at low glucose concentrations. The Beckman glucose analyser did not detect any difference in glucose concentration between the saline solutions and their protein-containing counterparts. It is unlikely that the effect is due only to the viscosity of the solution because the machine did not detect 'glucose' in a viscosity-adjusted flame photometer standard solution.

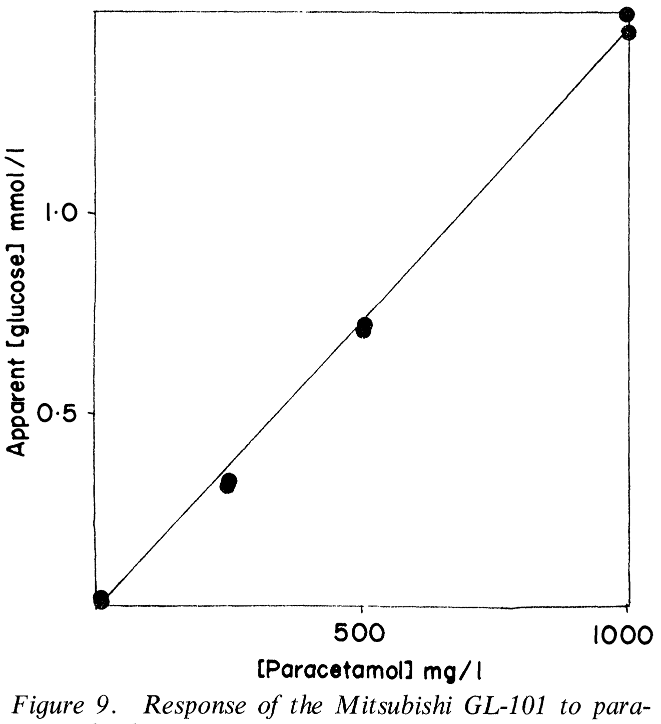

*Figure 9. Response of the Mitsubishi GL-101 to paracetamol solutions.*

*Table 2. Effect of protein on glucose results. Solutions containing 0, 1 or 5 mmol/l glucose in saline or saline containing protein were prepared and glucose concentrations measured on the Mitsubishi instrument. (HSA: human serum albumin.)*

| Prepared [glucose] (mmol/l) | Diluent | Mean measured [glucose] (mmol/l) n = 5 |
|---|---|---|
| 5 | Saline | 4·96 |
| | 72 g/l HSA in saline | 5·25 |
| 1 | Saline | 0·96 |
| | 72 g/l HSA in saline | 1·22 |
| 0 | Saline | 0·00 |
| | 80 g/l HSA in saline | 0·28 |
| | 40 g/l HSA in saline | 0·17 |
| | 50 g/l HSA + 30 g/l globulin in saline | 0·38 |

### Costs

To compare the costs of plasma glucose estimations on both machines only direct analytical costs were calculated since blood collection, separation of plasma, reporting of results and associated clerical work are common to both procedures. Costs were based on Good Hope annual work-load of approximately 15 000 samples; a number of assumptions, described below, were necessary.

Table 3 shows a breakdown of the expenses involved in running the Mitsubishi GL-101 and table 4 shows a similar analysis for the Beckman glucose analyser.

The capital cost per annum was based on a machine life of 10 years. The Beckman instrument was maintained on a fully inclusive service contract and the extra costs incurred for day-to-day maintenance were estimated at £150 per annum; however, the future maintenance costs of the Mitsubishi instrument were impossible to calculate and so were assumed to amount to 10% of the capital cost, as recommended by Broughton *et al.* [1].

Glucose oxidase reagent and pipette tips for the Beckman instrument were supplied by Beckman. Replacement columns and paper for the Mitsubishi machine are marketed by Anachem. Other reagent costs are for Fisons AR grade disodium hydrogen phosphate, potassium dihydrogen phosphate and benzoic acid and BDH 50 mmol/l glucose standard. Autoanalyser cups are supplied by BCL. The cost of distilled water was difficult to estimate, so the price quoted is based on the cost of BDH AnalaR water. Staff time was calculated from a salary of £6000 a year.

Despite the high capital cost of the Mitsubishi GL-101, the cost per test is lower on this machine (£0·18) than on the Beckman glucose analyser (£0·23) due to lower running costs. As a large proportion of the cost on the GL-101 is capital expenditure, the cost per test and the cost relative to the cost on the Beckman glucose analyser would be lower if the work-load were higher.

### Subjective evaluation

The GL-101 is simple to use and can be left unattended to complete the analysis of 40 samples on the sample turntable after which time it will automatically return to a standby condition. On completion of a run, the instrument emits a series of piercing bleeps for 30 s: these are very irritating and a single bleep would be adequate. The analyser is ideally suited to a heavy work-load of routine specimens, but several features detract from its use as an on-call instrument. If switched off, the instrument requires 30 min to warm up when reconnected, and if the power is left on

*Table 3. Breakdown of costs for the Mitsubishi GL-101 at an annual work-load of 15 000 samples.*

| | Unit cost | Cost per 1000 samples |
|---|---|---|
| *Capital and maintenance* | | |
| Capital | £8000 over 10 years | £53·33 |
| Maintenance | 10% of capital per annum | £53·33 |
| | | £106·66 |
| *Assay costs* | | |
| $Na_2HPO_4$ | £6·81 per kg | £0·31 |
| $KH_2PO_4$ | £9·70 per 500 g | £0·20 |
| 50 mmol/l glucose standard | £3·11 per 500 ml | £0·15 |
| Benzoic acid | £6·73 per 500 g | £0·10 |
| GOD column | £76 per pack of 5 | £6·08 |
| Pretreatment column | £76 per pack of 10 | £12·66 |
| Printer paper | £17 per 5 rolls | £0·68 |
| Water | £2·26 per 5 l | £6·75 |
| Analyser cups | £45·10 per 10000 | £4·51 |
| | | £31·44 |
| *Staff time* | | |
| Staff time | 45 min per day<br>Salary £6000 per annum | £43·92 |
| | | £43·92 |
| | Total | £182·02 |

*Table 4. Breakdown of costs for the Beckman glucose analyser at an annual work-load of 15 000 samples.*

| | Unit cost | Cost per 1000 samples |
|---|---|---|
| *Capital and maintenance* | | |
| Capital | £3785 over 10 years | £25·23 |
| Service contract | £500 per annum | £33·33 |
| Day-to-day maintenance | £150 per annum | £10·00 |
| | | £68·56 |
| *Assay costs* | | |
| Glucose oxidase reagent | £55·35/l<br>(pack includes standards) | £55·35 |
| Pipette tips | £20 per 1000 | £20·00 |
| | | £75·35 |
| *Staff time* | | |
| Staff time | 1·5 hours per day<br>Salary £6000 per annum | £87·84 |
| | | £87·84 |
| | Total | £231·75 |

it still performs a series of conditioning cycles for a period of up to 10 min. However, this is not adequate and if the machine has not been used for several hours, the results of the first tray are unreliable. It was necessary to run a short 'priming' tray of five standards and subsequently recalibrate the machine before analysing patients' samples.

During the time of the evaluation, several other problems were encountered. The first problem was the result of an air bubble in the electrode chamber. Once the bubble was dislodged and removed, the instrument performed well for several weeks. Further difficulties were met on the John Radcliffe instrument as a result of a blockage at the top of the pretreatment column. This was demonstrated by the junctions each side of the non-return valves becoming disconnected. As the block was in the column itself, the cause was not immediately obvious. Towards the end of the evaluation, problems were encountered with the temperature control in the pump chamber of the prototype instrument at the John Radcliffe Hospital. The fault was probably in the thermostat mechanism, since the temperature continued to increase although the circulation/cooling fan was operating. This malfunction was also associated with the production and accumulation of bubbles in the electrode chamber. The Good Hope instrument had no problems in this respect but developed a fault in the sample pump system. A microswitch controlling the travel of the syringe pump was maladjusted and failed to control the plunger movement. This resulted in the cessation of analysis, the activation of an alarm and the error message 'pump over-run'. The first time this occurred, Anachem were prompt in attending to the problem; however, when the problem recurred, they were unhelpful as they had no spare parts in the UK—a member of Good Hope's laboratory staff corrected the fault.

A most disconcerting problem was the production of one incorrect result in a sequence of answers, though this problem was much more pronounced on the prototype analyser. These spuriously low results were obvious when they appeared in tests of replication or correlation with an alternative technique. In the routine laboratory, they certainly would not become obvious. It was suggested that small air bubbles in the bottom cone of the sample cups might have resulted in insufficient sampling. If care was taken to remove any obvious air bubbles, performance on the Good Hope instrument was satisfactory, however, this explanation did not appear to account for all the spurious results from the prototype instrument.

The sample identification collars were of little help and the only ones used during the trial period were the standardization card and the end-of-run detector.

### Conclusions

The main impression was of a compact, well machined, simple-to-operate analyser, which generally performed well. The carry-over figure was acceptable and the correlation with the existing methods was reasonable, although the bias at low concentrations of glucose with one instrument gave cause for concern. The effect of other carbohydrates, ascorbic acid, urinary preservatives, fluoride, oxalate and paracetamol were within acceptable limits but grossly haemolysed specimens should be avoided. Despite the high capital cost of the instrument, the cost per test compared favourably with that of an alternative, widely-used instrument.

### Acknowledgements

The authors wish to thank both Mitsubishi and Anachem for the loan of one instrument. They also wish to thank the Bacteriology Department, Good Hope Hospital, for assistance with the microbiological safety tests and Mr R. A. Hall for his support of this project.

### References

1. Broughton, P. M. G., Gowenlock, A. H., McCormack, J. J. and Neill, D. W., *Annals of Clinical Biochemistry*, **11** (1974), 207.
2. Skinner, A. G., Hurrell, A. E., English, M. L., Clarke, N. V. and Johnstone, J. M., *Department of Health and Social Security Evaluation of the Burkard Econoflo Micro-Analytical System* (November 1980).
3. Broughton, P. M. G., Buttolph, M. A., Gowenlock, A. H., Neill, D. W. and Skentelberry, R. G., *Journal of Clinical Pathology*, **22** (1969), 278.
4. Caraway, W. T., In *Fundamentals of Clinical Chemistry*, Ed Tietz, N. W. (Saunders, 1976), p. 245.
5. *Handbook of YSI analyser* (Yellow Springs Instruments).

